# Secondary Immune Thrombocytopenia Associated With Asymptomatic COVID-19 Successfully Managed With Intravenous Immunoglobulin and Glucocorticoids

**DOI:** 10.7759/cureus.15153

**Published:** 2021-05-21

**Authors:** Hazem Ayesh, Azizullah Beran, Mohammed Mhanna, Srini Hejeebu

**Affiliations:** 1 Internal Medicine, The University of Toledo, Toledo, USA

**Keywords:** immune thrombocytopenia, covid-19, intravenous immunoglobulin, thrombocytopenia, glucocorticoid

## Abstract

Immune thrombocytopenia (ITP) is an autoimmune disease characterized by low platelet counts of <100 × 10^9^/L with the absence of other blood count abnormalities. Coronavirus disease 2019 (COVID-19) is caused by severe acute respiratory syndrome coronavirus 2 which is manifested by a severe multisystemic disease. We present the case of a 76-year-old female who presented with ITP associated with COVID-19 and successfully managed with intravenous immunoglobulin and glucocorticoids.

## Introduction

Immune thrombocytopenia (ITP) is an autoimmune disease characterized by low platelet counts of <100 × 10^9^/L with the absence of other blood count abnormalities [1]. ITP is classified as primary when history, physical examination, and laboratory results did not reveal apparent cause or secondary when it is associated with the underlying condition [1]. Coronavirus disease 2019 (COVID-19) is caused by severe acute respiratory syndrome coronavirus 2 (SARS-CoV-2) which is manifested by a severe multisystemic disease that can result in acute respiratory distress syndrome and multiorgan failure [2]. Thrombocytopenia is detected in 5-41.7% of COVID-19 patients and is typically mild (>100 × 10^9^/L) [3]. Thrombocytopenia in COVID-19 is associated with an increased risk of in-hospital mortality with lower platelets count associated with higher mortality [4]. Significant thrombocytopenia (<100 × 10^9^/L) is uncommon in COVID-19 [5], and very low platelets count of <20 × 10^9^/L in COVID-19 patients indicate immune etiology [5]. In this case report, we discuss a case of COVID-19-associated ITP managed successfully with glucocorticoids and intravenous immunoglobulin (IVIG).

## Case presentation

A 76-year-old female patient with a past medical history of insulin-dependent type 2 diabetes mellitus, essential hypertension, cerebrovascular accident (on aspirin and clopidogrel), and hyperlipidemia presented with a five-day history of skin rash that started on bilateral lower extremities and spread to the rest of the body. She also reported fatigue, mouth pain, visual disturbances, and arthralgia. Review of systems otherwise was unremarkable.

On presentation, she was alert and oriented. Vital signs showed blood pressure of 132/66, pulse rate of 63 beats/minute, temperature of 36.4°C, and respiratory rate of 18 breaths/minute. Physical examination revealed multiple hemorrhagic blisters over the soft palate and diffuse bilateral non-blanchable petechial rash. The rest of the physical examination was unremarkable. Notable labs showed low platelets at 3 × 10^9^/L, normal white blood cells at 5.9 × 10^9^/L, low hemoglobin at 7.6 g/dL, elevated international normalized ratio (INR) at 1.4 with normal activated partial thromboplastin time. COVID-19 reverse transcriptase-polymerase chain reaction came back positive.

**Figure 1:**
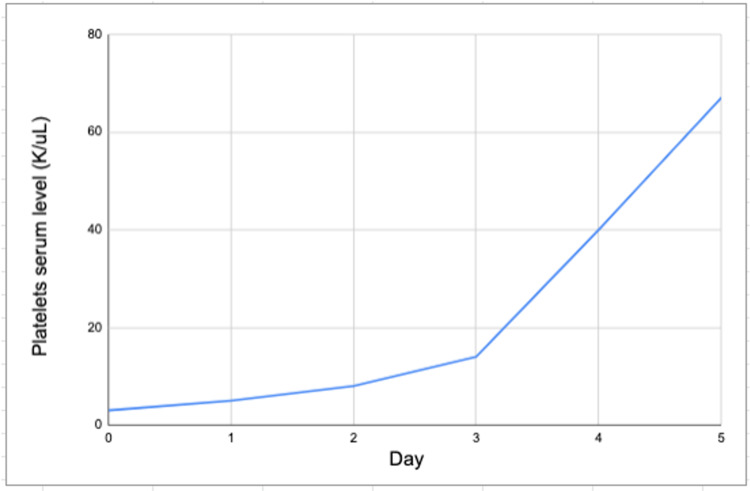
Increase in platelets level after initiation of IVIG and dexamethasone. IVIG: intravenous immunoglobulin

After initial stabilization, she received two units of platelet transfusions, and aspirin and clopidogrel were held. Hematology was consulted and they decided to start her on weight-based IVIG and dexamethasone burst therapy. Further diagnostic workup showed that hepatitis B, hepatitis C, and human immunodeficiency virus (HIV) panels were unremarkable. Serum protein electrophoresis showed polyclonal immune response, antinuclear (ANA) antibodies were positive, but anti-dsDNA and anti-smith antibodies were negative. Vitamin B12, Folate, iron, and thyroid-stimulating hormone (TSH) serum levels within normal limits. Abdominal ultrasound was unremarkable for splenomegaly. Peripheral blood smear showed low platelets with occasional large platelets. Factor VII activity was low normal at 54% (normal range: 50-129%), Factor V activity was normal at 93% (normal range: 62-139%), and Factor 10 activity was low at 57% (normal range: 70-150%). She completed two days of IVIG therapy and five days of dexamethasone 20 mg. Platelets level improved, as shown in Figure 1.

The patient was discharged in stable medical condition on vitamin K supplement and prednisone. INR improved to 1.2 on the day of discharge. Aspirin and clopidogrel was held on discharge, and follow-up with hematology clinic was scheduled.

## Discussion

ITP is a diagnosis of exclusion. In this patient, HIV, hepatitis B, hepatitis C, TSH, folate, vitamin B12, iron lab testing were all unremarkable. Splenomegaly was ruled out with abdominal ultrasound. Blood smear did not reveal atypical findings. ANA antibodies were positive, but anti-dsDNA and anti-smith antibodies were negative, and there were no strong clinical features supporting the diagnosis of systemic lupus erythematosus in this case. The fact that the patient had anemia with low hemoglobin may be explained by active bleeding. The reticulocyte index came back low at 1.02 which may indicate hypoproliferation. This finding is common in acute bleeding (less than seven days) as in our case. Hemolytic anemia was less likely as bilirubin was not elevated, Coombs test was negative, and there was no evidence of hemolysis on the blood smear. Elevated INR was likely secondary to vitamin K deficiency as vitamin-K dependent factors (VII and X) are lower than non-vitamin K-dependent Factor V.

On presentation, the patient had low hemoglobin, fatigue, bleeding blisters, and severe thrombocytopenia (defined as platelets <20 × 10^9^/L). Given the low platelets level and active bleeding, two units of platelets were transfused. Hematology decided to start a combination of IVIG and dexamethasone. The American Society of Hematology recommends either dexamethasone or prednisone as initial therapy in symptomatic patients and platelet count of <30 × 10^9^/L [6]. They also recommend adding IVIG if a more rapid response of platelets is required [6]. Recent practical guidelines published to address the management of ITP in COVID-19 patients suggested the use of glucocorticoid as initial therapy with a taper over two weeks [5]. The guidelines also recommended IVIG as a second-line in case of active bleeding or failure to respond to glucocorticoid [5]. The guidelines also reported that thrombopoietin receptor agonists (TPO‐RAs) such as eltrombopag may increase the risk of thrombotic complications in COVID-19 patients [5].

A recent systematic review involving 45 cases of new-onset ITP in COVID-19 patients reported that 22% of cases were treated with glucocorticoid alone, 29% were treated with IVIG alone, and 24.5% of cases were treated with a combination of glucocorticoid and IVIG [7]. A recent case series of 11 patients showed that three patients were managed with glucocorticoid alone, one patient was managed with IVIG alone, five patients were managed with a combination of glucocorticoid and IVIG, and two patients were managed with glucocorticoid and eltrombopag [8]. In summary, literature supports the use of combination IVIG and glucocorticoid as an early treatment modality in patients with active bleeding and severe thrombocytopenia. The role of utilizing TPO‐RAs in this patient group is unclear and may be associated with adverse thrombotic events [5].

## Conclusions

We discussed the diagnosis and management of secondary ITP associated with COVID-19. It is important to recognize ITP diagnosis early and differentiate it from COVID-19-associated thrombocytopenia to lower mortality and provide a more targeted therapeutic plan. We also highlighted practice differences in this population compared to patients without COVID-19. More standardized studies are required to evaluate the potential benefits and side effects of different treatment lines in COVID-19 patients.
